# Spanish Version of the Flourishing Scale (FS) on the Parents of Children With Cancer: A Validation Through Rasch Analysis

**DOI:** 10.3389/fpsyg.2019.00035

**Published:** 2019-01-25

**Authors:** Carmen Pozo Muñoz, Blanca Bretones Nieto

**Affiliations:** ^1^Department of Psychology, University of Almería, Almería, Spain; ^2^Psychosocial Intervention and Health (HUM-792) Research Group, University of Almería, Almería, Spain

**Keywords:** validation, rasch analysis, flourishing, parents, childhood cancer

## Abstract

The interest in the study of flourishing is due to the fact that it has been proven that it contributes to a good adjustment to the demands of the environment, relating to indicators of health and well-being. There are many researches that have tried to find out what dimensions make it up. The goal of this study is to validate the Spanish Version of The Flourishing Scale (FS), being the first time it is applied to Spanish parents of children with cancer. A total of 138 parents of children with cancer participated in a semi-structured interview. Through IBM SPSS and Winsteps descriptive analyzes and the internal consistency of the FS were calculated. Rasch analysis was used to study the dimensionality of the scale, the adjustment of each one of the items, the reliability values for the items and for the people, the validity of the construct, the functioning of the response categories, and the differential item functioning (DIF). The external construct validity of the FS was examined with associated measures. Results found that parents indicated a flourishing attitude (*M* = 3.85; *sd* = 0.63). We confirm that the FS maintains an adequate internal consistency and a unidimensional structure. We observed a good alignment between the question and the person's abilities as soon as a high reliability for the items. Although the sample is large enough to corroborate the difficulty hierarchy of the items, the construct validity could be improved by introducing items of moderate and high difficulty. On the other hand, the answer category 3 overlaps with the 2 and 4, so we propose possible solutions. Regarding a possible DIF, this exists in relation to “gender,” “level of education” and “by the situation with respect to treatment.” Finally, the convergent validity of the FS is demonstrated, there being a significant correlation with well-being, satisfaction with social support and coping. In general, the results show adequate psychometric properties of the Spanish version of the FS, so we recommend integrating it in psychosocial interventions aimed at parents of children with cancer, in order to provide them with resources to deal with the disease.

## Introduction

The central concept developed by Keyes (2007) defines the flourisher as the individual who experiences a high level of emotional, psychological and social well-being. In subsequent studies, Diener et al. (2010) consider that the flourishing consists of a sense of competence, self-acceptance, optimism, and to contribute to the well-being of others. According to Huppert and So (2009) flourishing comprises a set of central characteristics including: positive emotions, delivery, interest, meaning and purpose; and an additional set of competencies: self-esteem, optimism, resilience, vitality, self-determination, and positive relationships.

Well-being Theory is based on the Theory of Authentic Happiness, centered on the study of the latter to increase satisfaction with life. However, from the theory of well-being, positive psychology focuses its interest on the analysis of it, using flourishing for its measurement (Seligman, 2011). Flourishing has been related to psychological well-being and has been studied in conjunction with positive and negative emotions in order to elaborate scales to help us understand well-being (Diener et al., 1999).

Agreement has not been reached regarding the dimensions that comprise the Flourishing Scale. The first results come from the application of the original Flourishing Scale (FS) by Diener et al. (2009). The FS scale was found to have a single strong factor with an eigenvalue of 4.24, which accounted for 52% of the items' variance, with Cronbach's alpha of 0.87 and a test-retest reliability of 0.71.

Subsequent studies have obtained similar results. In Brazil, Nunes et al. (2015) support the one-dimensionality of the scale in two studies carried out, respectively with students and the general population. Similarly, Tang et al. (2016) obtained a single factor in a sample Chinese population (α > 0.90 and a good convergent and discriminant validity); as did Schotanus-Dijkstra et al. (2016) in research conducted with adults in the Netherlands (α = 0.86; adequate global reliability and a good fit of most of the elements in the Rasch analysis).

Recent studies in Spain have applied the Spanish version of the FS. In a study by Ramírez et al. (2017), of university students and patients with chronic back pain, the main axis factor, and the simultaneous component analysis in both groups showed a common unidimensional structure. In addition, the omega coefficient showed high reliability. The principal conclusion was that flourishing had a mediating effect between personality variables and positive and negative affect, which explains the association between anxiety, optimism, pessimism, and positive affect.

De la Fuente et al. (2017) have also supported the Spanish version of the FS as a suitable measure. Specifically, they applied it with university students from different fields of knowledge. They obtained a single factor structure of the FS, with good internal consistency. The convergent validity of the scale was demonstrated with another measure of psychological well-being and its discriminant validity with the scale of symptoms of depression, anxiety, and stress.

It is worth highlighting the cross-cultural research of Pozo et al. (2016), in which Colombian and Spanish university students participated. Satisfactory psychometric properties (α Colombian = 0.88; α Spanish = 0.85) and good levels of reliability were found (the Rasch person reliability was fairly acceptable -Colombia = 0.77; Spain = 0.80-, and the person separation statistic was 1.83 for Colombia and 2.02 for Spain. Rasch item reliability was 0.93 for Colombia and 0.94 for Spain, and the item separation statistic was 3.69 for Colombia and 4.13 for Spain. In both samples, the Cronbach alpha values were higher than 0.70).

Giuntoli et al. (2017) analyze the dimensionality of the construct (they found a superior fit for a two factor model) and its invariance in two studies (with students, unemployed and healthy samples) through a multigroup analysis. Concurrent validity was verified with other well-being, depression and anxiety measures.

In relation to the fields of application, previous investigations have verified the association between flourishing and less limitations in daily life, as well as flourishing and better health (Howell et al., 2013; Huppert and So, 2013; Gilmour, 2015). Along the same lines, the psychosocial factor in the context of education has been studied and found to be beneficial regarding the well-being, academic performance, and abilities of university students (Fredrickson and Branigan, 2005; Howell, 2009; Wilson-Strydom and Walker, 2015), as well as for occupational health workers (Rautenbach and Rothmann, 2017). Recently, flourishing has been analyzed as a positive coping mechanism for health and well-being in the context of childhood cancer, specifically in the parents of diagnosed children and adolescents (Bretones et al., 2016; Bretones, 2018).

Although the psychometric properties of FS have previously been studied through the Classical Test Theory (CTT) (Diener et al., 2009; Silva and Caetano, 2013; Hone et al., 2014; Sumi, 2014), on a few occasions analysis of the psychometric properties of this instrument has been used by new models of the Item Response Theory (IRT), such as the Rasch model (Pozo et al., 2016; Schotanus-Dijkstra et al., 2016).

The main advantages of the Rasch Model (the most parsimonious IRT model) in comparison with CTT are the possibility of estimating the extent to which each test or item measures the skill of the participants, the joint estimates of respondents' parameters and items, and the invariance of the parameters obtained from the different samples (Arias et al., 2013; Garzón Umerenkova et al., 2017). In contrast to the CTT, the Rasch model establishes the probability of a person's response to a stimulus, i.e., the difference between the measure of a person's trait and the measure of the stimulus used (Tristán, 2002). Likewise, the Rasch model quantifies the amount of information and error with which it is measured in each point of the dimension and allows the selection of those items that make it possible to increase the information in previously specified regions of the construct (Jiménez and Montero, 2013).

In light of the above, it has already been supported that a flourishing attitude is related to well-being indicators. Childhood cancer is understood as a “family disease” because it affects the health of all its members and determines its ongoing functioning (Bretones, 2018). The importance of analyzing the psychosocial repercussion of this pathology in the parents is due to the fact that they have been considered as “invisible patients” (Hinds, 1992). On the other hand, it has been shown that the adaptation of children and adolescents is determined by factors related to the degree of adjustment of their parents, especially in the initial stages of the disease (Fedele et al., 2013). During the childhood illness the parents of children with cancer have to suffer a lot of critical moments in relation with diagnosis, treatments, side effects, surgeries, e.g., In this way, stress symptoms represent a common experience in the familiar context, also after the illness situation (Vrijmoet-Wiersma et al., 2010). Taking into account these circumstances and since flourishing has previously been related to variables related to health and well-being in the context of illness (Bretones et al., 2016), we consider it important to verify the effects that Flourishing causes in terms of coping with the disease. For this, first of all we believe that it is necessary to continue deepening the analysis of the Flourishing Scale, as a novelty, in a sample of Spanish parents of children with cancer.

### Objectives

The purpose of this study was to validate the Spanish version of the Flourishing Scale following administration in a sample of parents of children with cancer. This was done using Rasch analysis in particular, also dimensionality, the fit of the items to the model, the functioning of the measurement scale, construct validity, reliability, and the differential item functioning (DIF). Finally, the convergent validity of the FS was determined in relation to other measures that study the following variables: well-being, coping styles and social support (these variables have been chosen because they represent indications of the state of health and quality of life of the parents of children with cancer, in this case).

## Methodology

### Sample

The sample consisted of 138 adults: 60 men and 78 women, who were the parents of 94 children (aged between 1 and 14 years) diagnosed with cancer. The parents' ages ranged from 41 to 50 years old 0.84% were of Spanish nationality and 16% of foreign origin (10% African and 6% Romanian). In this cohort 88.4% were married or living together as a couple, 60.2% had completed secondary school and 53.9% worked in the services sector (includes subsectors such as commerce, communications, call center, finance, tourism, hospitality, leisure, culture, entertainment, public administration, and so-called public services, provided by the State or private initiative (health, education, care dependency; among others). The diagnoses of the children were: leukemia (44.2%) and solid tumors (55.8%).

### Measures

This study comprises a quantitative part (on which this article focuses, with validated Likert-type scales) and a qualitative part (a series of open-ended questions designed to investigate the main problems in fundamental areas of the family's daily life) of the data gathered during the semi-structured interviews in which they evaluated: socio-demographics and clinical characteristics, objective and subjective health, well-being, and stressors, the majority used coping strategies (including flourishing), as well as support from their formal and informal network.

Because of the characteristics of the study, sociodemographic and clinical variables of interest were collected. Socio-demographics included: age, gender, nationality, educational level, marital status, and employment situation. Regarding the children, clinical information was required. These were: the diagnosis, date of diagnosis, type of treatment and current status post-treatment.

The adaptation of the Symptom Scale (Martos et al., 2009) consists of a 9-point Likert scale that assesses perceived physical and psychological symptoms. Subjective health was assessed with the item “*In general, I am in poor health.”* The internal consistency in the original scale, according to Cronbach's alpha statistic is 0.79 (Jou and Foucada, 1997), whereas in the present study it is 0.85.

The Spanish version of the Satisfaction with Life Scale (Goodwin and Hernández, 2000) is a 5-item Likert scale used to assess the level of well-being experienced by parents. Answers range from 1 (completely disagree) to 5 (completely agree). Cronbach's alpha in the original scale is 0.87 (Diener et al., 1985), while it is 0.82 in this study.

In order to analyze the coping styles in parents, the reduced Spanish version of the Cope Inventory (*Cuestionario de Afrontamiento del Estrés*, CAE) (González and Landero, 2007) was applied. This scale consists of 21 items, which are divided into seven subscales (although the total score of the scale was used): problem-solving coping, negative auto-focused coping, positive reappraisal, overt emotional expression, avoidance coping, social support seeking, and religious coping. Items are answered on a 5-point Likert scale ranging from 0 (never) to 5 (almost always). While Cronbach's alpha for Sadín's and Chorot's original questionnaire reached 0.77, in CAE's reduced version internal consistency was 0.74, and finally, in this study it reached 0.62.

The flourishing attitude was evaluated through the Spanish version of the Flourishing Scale (original by Diener et al., 2009). This scale is made up of eight items. The values of the answers range from 1 to 5 (1 being “strongly disagree”; 2 “disagree”; 3 “neither agree nor disagree”; 4 “agree” and 5 “totally agree”). Cronbach's alpha in the original scale is 0.87 (Diener et al., 2009), whereas it is 0.74 in the present study.

### Procedure

The study was presented to and approved by the Research Ethics Committee of Torrecárdenas Hospital (Almeria, Spain). Research methodology was planned considering the design of the inclusion and exclusion criteria, the sample selection, the interview design, etc. The requirements for participation in the interview were being the parent of a child diagnosed with cancer who had been treated in that hospital. Exclusion criteria was determined as the following: being the parent of a child who had died due to the disease and a parent who did not speak Spanish with sufficient command.

The interview was drawn up according to the research objectives and administered individually (a single session) in a private room of the hospital (approximately one hour's duration), following detailed explanation of the study's characteristics. Participants signed consent forms and all data was treated in accordance with current privacy laws. These were recorded and transcribed verbatim to a text file. Audio and text files were then saved and processed confidentially.

### Data Analysis

The results were analyzed using IBM SPSS software (version 22 for Windows) and Winsteps (v. 3.72.3) statistical package. The following analyses were conducted:

– Descriptive analyses.
– Internal consistency of the items calculated using Cronbach's alpha.
– The unidimensionality of the test was verified by “main components.”
– The goodness of fit of each of the eight FS items (infit and outfit estimates) to the Rasch model.
– Reliability values were calculated for the items and for the subjects.
– The validity of the construct was verified based on the hierarchy of the items (Wright map).
– The functioning of the response categories was verified through the Rating Scale Model (RSM) for polytomous items.
– The possible differential item functioning (DIF) was contrasted.
– Finally, FS construct validity was examined with a wide variety of measures. Pearson correlation coefficients were used to evaluate the convergent validity between the Spanish version of the Flourishing Scale (FS), the Spanish version of the Satisfaction with Life Scale, the reduced Spanish version of the Coping Stress Questionnaire (CAE) and the adaptation of the Perceived Social Support Scale (EASP/PSS).

## Results

### Descriptive Analysis

The results exceed the midpoint of the scale (*M* = 3.85; *sd* = 0.63). This also happens in specific items, such as being considered a “good person and lead a good life,” feeling respected, subjectively perceived as “competent and capable,” contributing to the happiness and well-being of people close to them and having a “useful and meaningful” life (Table 1).

**Table 1:** Items of the Spanish Version of the FS.

**Ítems**	**$\bar{\text{x}}$ ± sd**
I am a good person and live a good life (item 6)	4.48 ± 0.81
People respect me (item 8)	4.41 ± 0.87
I am competent and capable in the activities that are important to me (item 5)	4.28 ± 1.10
I actively contribute to the happiness and wellbeing of others (item 4)	4.25 ± 1.03
I lead a purposeful and meaningful life (item 1)	4.11 ± 1.08
I am engaged and interested in my daily activities (item 3)	3.76 ± 1.25
I am optimistic about my future (item 7)	3.62 ± 1.24
My social relationships are supportive and rewarding (item 2)	1.91 ± 0.95

On the other hand, defining oneself as optimistic and being involved in daily activities was also considered. Despite this, the lowest scores are related to parent's perceptions about their supportive and rewarding social relations.

### The Rasch Model on the Flourishing Scale

Cronbach's alpha for the Spanish version of the FS was 0.74. In this sense, it is possible to conclude that it is structured in a single dimension. The variance explained by the measure was 59.2%, with a first contrast *eigenvalues* of 1.6, meeting the recommendations for assuming the dimensionality (variance > 0.30; first contrast eigenvalue < 3.0) stated by of Bond and Fox (2007, 2012) or Smith (2012). The item fit to the Rasch Model of each of the eight items of the scale was checked. All items were between 0.5 and 1.5 (the MNSQ values of Infit and Outfit) (see Table 2).

**Table 2:** Infit and outfit estimations for each item.

			**INFIT**	**OUTFIT**	**PT-MEASURED**	
**Item**	**Measure**	**Model SE**	**MNSQ**	**MNSQ**	**CORR**.	**EXP**.
Item7	0.35	0.09	1.37	1.40	0.49	0.65
Item2	2.30	0.10	1.16	1.36	0.48	0.57
Item6	−0.88	0.12	1.11	0.97	0.48	0.52
Item5	−0.50	0.11	1.04	0.79	0.66	0.57
Item1	−0.25	0.10	1.03	1.02	0.60	0.60
Item8	−0.75	0.12	0.96	0.98	0.54	0.54
Item4	−0.46	0.11	0.89	0.75	0.65	0.58
Item3	0.19	0.09	0.83	0.81	0.73	0.64
Item mean	0.00	0.11	1.05	1.01	–	–
SD	0.96	0.01	0.16	0.23	–	–

*The order of the items corresponds to the adjustment of each of the items according to the output of the table in the Rash model*.

*Measure, Location of the latent variable; Model SE, Error estándar; INFIT-MNSQ, Infit Mean Squared; “OUTFIT-MNSQ, Outfit Mean Squared; PT Measured, Point Measure Correlation*.

The PT-Measure-CORR column allows observation of the alignment between the question and the subject's abilities. The results indicate that there are no inverse correlations and the associations are moderate (the lowest value is set in item 7, with a correlation of 0.49, and the highest item being 4, with a value of 0.73). In addition, columns PT-Measure-EXP. show that there are no notable differences between observed and expected correlations.

**Figure 1:**
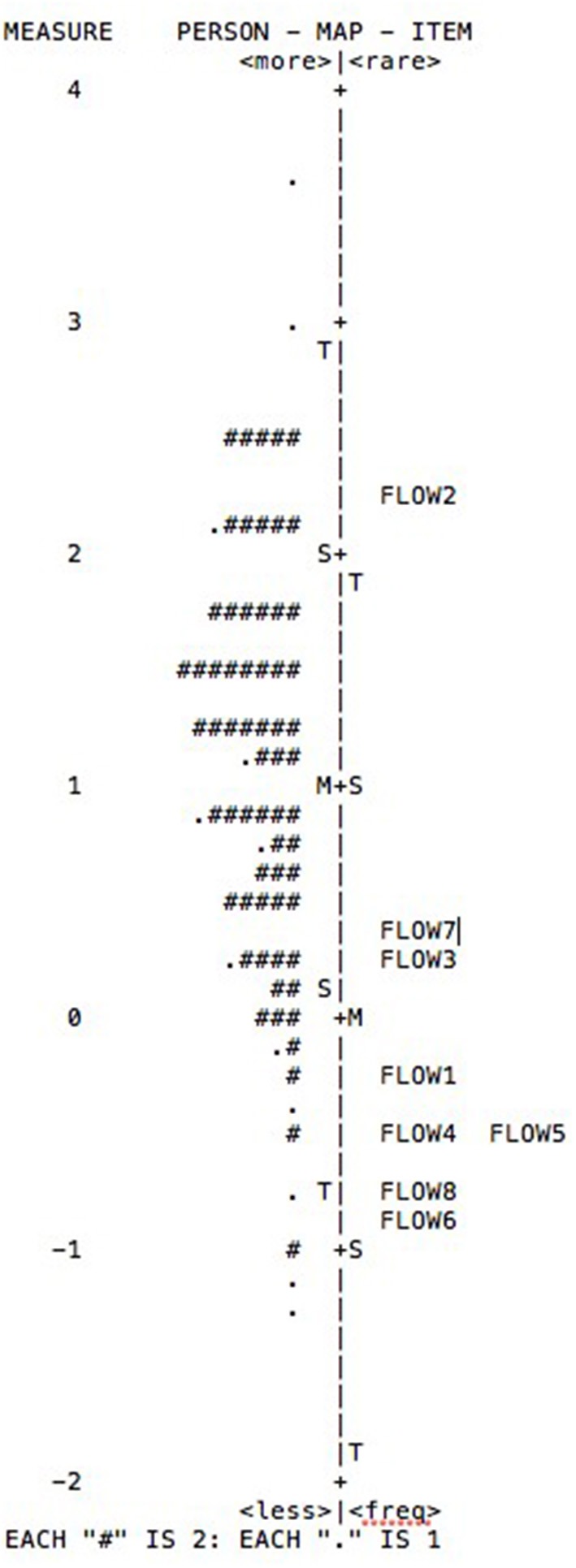
Wright map of person and items.

On the other hand, the reliability for the items was 0.99 with a separation of 8.58, with high values. The values of separation are >3. In relation to the reliability for people, a value of 0.65 and a separation of 1.36 was obtained. The next step was to analyse the construct validity based on the hierarchy of the items using the Wright map (Figure 1). Unlike the results obtained in other studies (Pozo et al., 2016), in this case, there was a number of subjects in the center of the figure who do not have items in front of them. They are at a moderate level of flourishing, but the scale does not have adequate items to measure the level of the variable.

**Figure 2:**
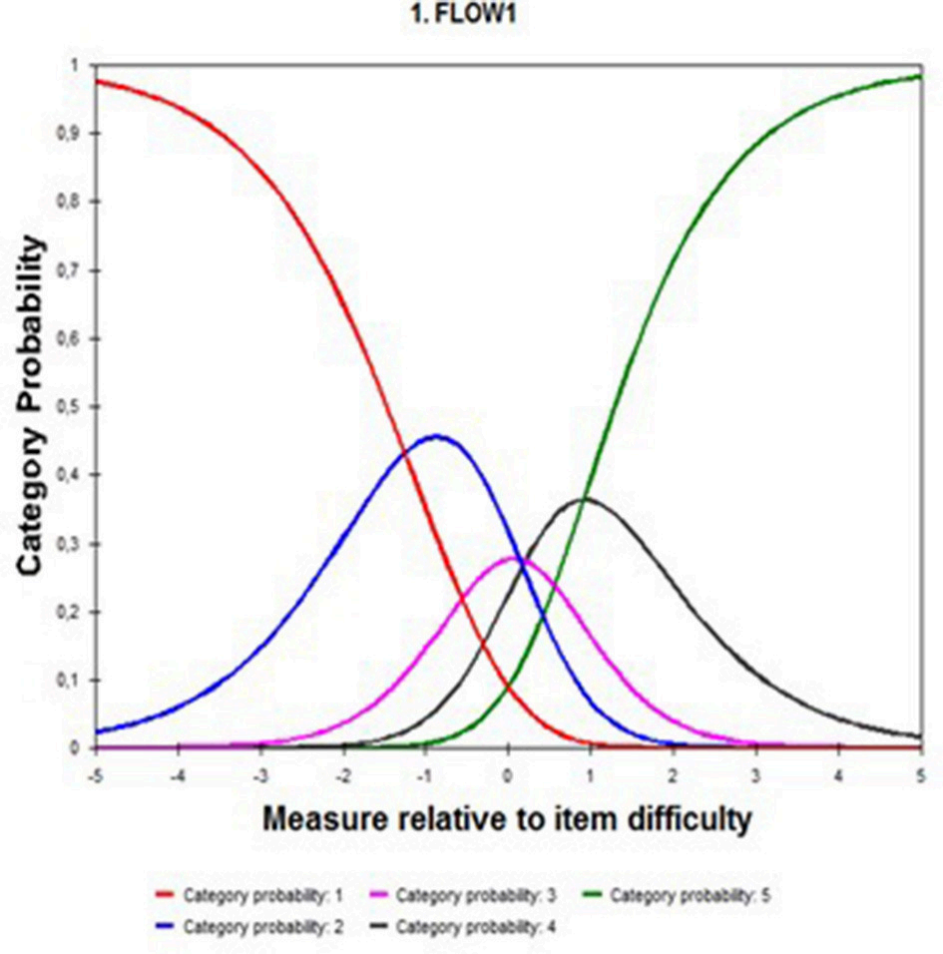
Probability curves for the response categories.

Response categories' function was studied through the Rating Scale Model (RSM) for polytomous items. The response categories were the following: (1) means “strongly disagree”; (2) “somewhat disagree”; (3) “neither agree nor disagree”; (4) “somewhat in agreement,” and (5) “totally agree.” In the probability curves of the categories it was found that category 3 overlapped with categories 2 and 4 (see Figure 2).

The possible Differential Item Functioning (DIF) of the items was checked by “gender,” “educational level” and “the child's situation with respect to treatment.” According to Prieto et al. (2011), an item has a DIF associated with belonging to a group when people with the same value in the measured variable, but from different groups, have a different probability of solving that item correctly. In this way, it was established as a criterion for a possible DIF that the values of “DIF contrast” were >0.5 logits between the comparison groups and that this difference in the t values was significant (*p* ≤ 0.05; Bond and Fox, 2007, 2012).

A possible DIF by “gender” was found for item 7 *(“I am optimistic about my future”)* with a contrast of 0.62 (*p* ≤ 0.05), being a more difficult item for women than for men. There was also a possible DIF by “level of education” for item 5 *(“I am competent and capable in the activities that are important to me”)*, with a contrast of 1.41 (*p* ≤ 0.05), being a more difficult item for participants without studies than for those with higher education.

It is equally important to consider a possible DIF with “the child's situation with respect to treatment” for item 6 *(“I am a good person and live a good life”)*, with a contrast of 0.84 (*p* ≤ 0.05). It is a more difficult item for parents whose children are receiving treatment, compared to participants whose children have been “out of treatment” for >5 years.

Finally, regarding convergent and divergent validity, our results largely confirmed the correlations between FS and other measures under study with a Bonferroni-adjusted (Table 3). We found a moderate relationship between flourishing and well-being (*r* = 0.403, *p* ≤ 0.01). The FS also showed a low correlation with the satisfaction with the social support received (*r* = 0.293, *p* ≤ 0.01) and with coping strategies that parents use; specifically, “the positive re-appraisal” (*r* = 0.211, *p* ≤ 0.05); with “the problem-solving coping” (*r* = 0.186; *p* ≤ 0.05), the “negative auto-focused coping” (*r* = −0.248; *p* ≤ 0.01) and the “over emotional expression” (*r* = −0.171; *p* ≤ 0.05).

**Table 3:** Correlation between Flourishing and other measures.

	**1**	**2**	**3**	**4**	**5**	**6**	**7**
1. Flourishing	1	–	–	–	–	–	–
2. Wellbeing	0.403**	1	–	–	–	–	–
3. Satisfaction with social support	0.293**	0.099	1	–	–	–	–
4. Positive re–appraisal	0.211*	0.265**	0.137	1	–	–	–
5. Problem–solving coping	0.186*	0.158	0.010	0.177*	1	–	–
6. Negative auto–focused coping	−0.248**	−0.188*	−0.029	-0.328**	-0.161	1	–
7. Over emotional expression	−0.171*	−0.057	−0.111	-0.043	-0.008	0.247**	1

* p ≤ 0.05;

** p ≤ 0.01;

## Discussion

The purpose of the present study consisted of validate the Spanish version of the Flourishing Scale following administration in a sample of parents of children with cancer. As a first step, descriptive analyzes were carried out to check the tendency of the sample to show a flourishing attitude. If were considered the results of this study with those obtained in other investigations in which other groups (specifically, university students) have contributed (Pozo et al., 2016; Garzón Umerenkova et al., 2017) it is possible to affirm that the parents who have participated in this study have a flourishing attitude, because the scores obtained exceed the midpoint of the scale. Good results are maintained with regard to the internal consistency of the Spanish version of the FS.

The findings of the Rasch analysis have shown the one-dimensionality of the scale and there is a good fit to the model of each of the eight items that make up the scale.

The infit and outfit mean-square (MNSQ) statistics provide evidence of construct validity when expected values are close to 1.0, with values from 0.5 to 1.5 being useful for measure, so in this case a good fit to the model is confirmed. Equally, there is a good alignment between the questions and the skills of the respondent.

In accordance with Smith (2012), the Rasch model verifies that the sample is large enough to corroborate the difficulty hierarchy of the items, namely, the construct validity of the instrument the reliability of people is considered a low index of separation for people with inferior values to 2. Therefore, the instrument is not as sensitive as one would wish, for identifying people with high and low skill. The differences in this study compared to other studies may be due to the diversity of the characteristics of the samples used (Pozo et al., 2016; Garzón Umerenkova et al., 2017).

The construct validity of FS can be improved by adding items of moderate and high difficulty. This would increase the sensitivity of the instrument and the reliability for people, since these two points are related.

The overlap found between the response categories could be solved by eliminating category 3, reducing the Likert scale from five to four points, or the term “neither agree nor disagree” could be rewritten, which would seem to be insufficiently differentiated from the terms “somewhat in disagreement” (2) or “somewhat in agreement” (4).

Originally called “item bias” (Lord, 1980), Differential Item Functioning (DIF) has been recognized as a potential source of bias in person measure. However, other authors indicate that the existence of the DIF does not necessarily imply a bias in the item (Wright and Stone, 1999). When analyzing the differential behavior of items (DIF), the results indicate a possible DIF by gender, by level of education and by situation with respect to treatment. In short, the aforementioned items should continue to be investigated in order to corroborate if there really is a bias or not.

## Conclusion

As a strength, the Rasch analysis performed after the application of the Spanish version of the FS on the parents of children with cancer, indicates that generally the test presents a good psychometric performance. However, it would be appropriate to continue investigating the previously mentioned aspects to further improve its performance.

In relation to the convergent validity, our results are in agreement with the postulates of well-being Theory in the field of Positive Psychology (Seligman, 2011), since there is a moderate correlation between flourishing and perceived well-being. For this reason, it is important to continue researching, using flourishing as a measure. In this connection, in principle, we could interpret that flourishing is an effective coping strategy for the parents of children with cancer, because it is positively related to styles considered healthy (“satisfaction with social support,” “positive re-appraisal” and “problem-solving coping”) and in a negative way with others, that are not (“negative auto-focused coping” and “over emotional expression”). Therefore, it is possible to affirm that flourish people are more likely to develop more favorable behavior patterns in situations that may be problematic (such as the experience of having a child with cancer). It would be convenient to take these results into account in order to develop psychosocial intervention programs (planning actions that stimulate the development of Flourishing components) aimed at responding to the needs of the parents of children with cancer.

On the other hand, regarding the advantages of an IRT analysis with respect to the usual CCT, the use of the characteristics of the IRT method has constituted an advance for this study, allowing the improvement in the measurement of the scale as well as its refinement.

It is worth highlighting some methodological limitations detected that should be taken into account in subsequent studies (low separation, no items at a mid-range of the construct, some problem with the response scale, moderate correlations with validation measures). On the other hand, due to the number of cases of childhood cancer in the study context, it has not been possible to apply a specific sampling method. In addition, a large volume of experimental mortality has been observed. Regarding this issue, it has been found that research related to “childhood cancer” is a difficult field to gather large samples (Bragado et al., 2008). The language barrier (in the immigrant population) and the families that declined the invitation to participate, were the greatest difficulties. This is associated with the fact that, for most of the parents, submitting to the interview took great emotional effort, due to having to exercise awareness and delve into circumstances of their life that were especially painful for the child, the parents themselves, and the rest of the family.

Finally, the importance of these results makes it necessary to replicate this study in a population with similar characteristics in order to contrast the resulting conclusions and implement methodological improvements in relation to the scale. We even propose for a future investigation to explore through a pair of pairs of parents of the same child, so that both can individualize the different strategies to face the childhood illness on the part of the mother and the father, isolating this problem from the others. On the other hand, a global approach based on dyads could be useful in this case (even for correlation with other scales and the Rasch analysis) and could be useful to complement the results obtained.

## Ethics Statement

This study has been developed in accordance with the Declaration of Helsinki and has been approved by the Research Ethics Committee of Torrecárdenas Hospital (Almeria, Spain). There was no conflict of interest between the authors and other institutions.

## Author Contributions

CP was the researcher in charge of coordinating research planning, reviewing the literature, and subsequently proposing the objectives and research design. In addition, she has participated in the analysis phase of the data and in the writing of the article.

BB was responsible for selecting the final sample of parents who participated; she conducted the interviews, participated in the analysis phase of the data and in the writing of the article.

### Conflict of Interest Statement

The authors declare that the research was conducted in the absence of any commercial or financial relationships that could be construed as a potential conflict of interest.
